# Age Effects on Hypocotyl Mechanics

**DOI:** 10.1371/journal.pone.0167808

**Published:** 2016-12-15

**Authors:** Friederike Saxe, Susann Weichold, Antje Reinecke, Jan Lisec, Anett Döring, Lutz Neumetzler, Ingo Burgert, Michaela Eder

**Affiliations:** 1 Department of Biomaterials, Max-Planck-Institute of Colloids and Interfaces, Potsdam, Germany; 2 Cluster of Excellence »Image Knowledge Gestaltung«, Humboldt University, Berlin, Germany; 3 Plant Cell Wall Group, Max-Planck-Institute of Molecular Plant Physiology, Potsdam, Germany; 4 Charite´-Universitätsmedizin Berlin, Molekulares Krebsforschungszentrum (MKFZ), Berlin, Germany; 5 targenomix GmbH, Potsdam, Germany; 6 Institute for Building Materials, Federal Institute of Technology, Zurich, Switzerland; 7 Applied Wood Materials Laboratory, Swiss Federal Laboratories for Materials Science and Technology, Duebendorf, Switzerland; Universidade de Lisboa Instituto Superior de Agronomia, PORTUGAL

## Abstract

Numerous studies deal with composition and molecular processes involved in primary cell wall formation and alteration in *Arabidopsis*. However, it still remains difficult to assess the relation between physiological properties and mechanical function at the cell wall level. The thin and fragile structure of primary cell walls and their large biological variability, partly related to structural changes during growth, make mechanical experiments challenging. Since, to the best of our knowledge, there is no reliable data in the literature about how the properties of the fully elongated zone of hypocotyls change with age. We studied in a series of experiments on two different seed batches the tensile properties the region below the growth zone of 4 to 7 day old etiolated *Arabidopsis* hypocotyls. Additionally, we analysed geometrical parameters, hypocotyl density and cellulose content as individual traits and their relation to tissue mechanics. No significant differences of the mechanical parameters of the non-growing region between 5–7 day old plants could be found whereas in 4 day old plants both tensile stiffness and ultimate tensile stress were significantly lower than in the older plants. Furthermore hypocotyl diameters and densities remain almost the same for 5, 6 and 7 day old seedlings. Naturally, hypocotyl lengths increase with age. The evaluation whether the choice–age or length—influences the mechanical properties showed that both are equally applicable sampling parameters. Additionally, our detailed study allows for the estimation of biological variability, connections between mechanics and hypocotyl age could be established and complement the knowledge on biochemistry and genetics affecting primary plant cell wall growth. The application of two different micromechanical devices for testing living *Arabidopsis* hypocotyls allows for emphasizing and discussing experimental limitations and for presenting a wide range of possibilities to address current and future questions related to plant cell wall mechanics, synthesis and growth in combination with molecular biology methodologies.

## Introduction

The plant cell wall directs and determines plant cell and organ shape and provides mechanical stability despite other functions like the defence against pathogens, protection against dehydration and cell-cell-interactions. Chemical composition, molecular processes and the internal structure of cell walls influence their mechanical properties. In turn, the determination of mechanical properties gives insight into cell wall properties and growth processes of primary plant cell walls [1–4].

*Arabidopsis* hypocotyls represent an excellent system to investigate mechanical properties of living plant cells. If grown in the dark, the seedlings become etiolated and have the shape of long and slender cylinders without significant cell division or differentiation during growth [5]. Due to this geometry, the hypocotyls are experimentally well accessible for structural and mechanical investigations. Currently, the highly active and dynamic field of molecular biology creates numerous *Arabidopsis thaliana* mutants with various changes in cell wall composition and structure [6]. These mutants are of high value for further studies to explore the impact of alterations on structure, which directly affect mechanical properties [2, 7–10].

From experiments on different plant systems, mechanical properties are known to be strongly determined by structural cell wall features [2, 7, 11–13]. However, basic data about how mechanical properties of the elongated zone of *Arabidopsis* hypocotyls change with time are still missing. This basic data is important for systematic comparative studies of the fully elongated region of wild-type and mutant *Arabidopsis* hypocotyls.

*Arabidopsis* hypocotyls are comparably small and fragile which makes their structure difficult to investigate and mechanical experiments challenging [2, 8, 14]. Additionally, inherent biological variability is large and not fully understood but can be partly related to structural changes during growth processes: it is known that cell elongation of etiolated *Arabidopsis* hypocotyls starts with a slower growth phase followed by a rapid elongation along a steep acropetal gradient in the longitudinal direction [5, 15]. Furthermore cell wall thickness is not uniform, it rather depends on position and the developmental stage of a cell [16].

We studied the mechanical behaviour during tension of the lower part of 4, 5, 6 and 7 day old etiolated *Arabidopsis* hypocotyls (accession Col-0) with two different experimental setups. Based on the experimental data, tensile stiffness was calculated in the linear region of the stress-strain curve. The determined tensile stiffness is not a true Youngs Modulus or modulus of elasticity, since the hypocotyls are not ideally elastic [7]. Furthermore, ultimate tensile stress σ_max_, the highest stress value during an experiment which is typically reached when the sample starts to fracture, was determined. For data interpretation geometrical parameters, density and cellulose content were taken into account. All the experiments were focussed on the lower parts below the growth zone of the hypocotyls with the main goal to create a reliable database for future experiments on modified hypocotyls.

## Materials and Methods

### Growing conditions of hypocotyls

Seeds of *Arabidopsis thaliana* (L.) Heynh. (Col-0) were stored at 6°C before sterilization. They were then plated in 8.8 g/l Murashige and Skoog basal medium in 0.8% agar. Afterwards all plates were incubated in the dark at 22°C for 4, 5, 6 and 7 days respectively. For a tensile testing series 15–20 hypocotyls were analysed so that 45–60 hypocotyls were required for three repetitive measurements.

### Experimental setup 1: Microtensile tests in water saturated air

**Fig 1:**
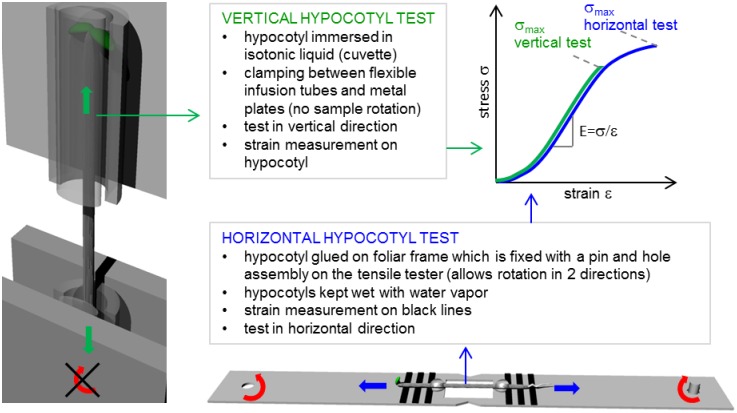
Clamping of hypocotyls in microtensile testing devices and schematic stress-strain curve.

For the experiments two seed batches (1 and 2, Col-O) were used to account for individual genetic variation. The seed batches were pooled from a variety of individual parent plants and seed batch 1 and 2 were grown at different times. Each seed batch was divided into 2 experimental series (a and b). The hypocotyls were tested at 4, 5, 6 or 7 days +/- 1.5 hours after sowing with a microtensile testing device which has been described in [17, 18]. The setup allowed a reasonably fast determination of mechanical properties (stiffness and ultimate tensile stress) along the longitudinal axis of the hypocotyl. After the length and diameter of a hypocotyl were measured by light microscopy, it was glued onto a foliar frame by using a combination of cyanacrylate glue and dental cement [7], Fig 1, horizontal hypocotyl test). A 2 mm long region in the lower part of the hypocotyl with fully elongated cells was chosen as the region to be tested to exclude the influence of different elongation states in the growth zone at the top of the hypocotyl [5, 15, 16] on the mechanical properties. After hardening of the glue (~10 min) the prepared samples were mounted with a pin-hole-assembly onto the tensile tester. The pin hole-assembly allows slight rotation of the sample in 2 dimensions (red arrow in Fig 1), allowing for sample alignment. Black lines close to the glue spots attaching the hypocotyl to the foliar frame served as video extensometry markers for strain determination. The samples were strained with a speed of 10 μm/s, force was recorded with a load cell with a maximum capacity of 500 mN (Honeywell Sensotec, Model 31E). To avoid drying during the test, the hypocotyls were rinsed with water vapour applied by a 1 mm diameter pipe from a commercial air humidifier.

### Experimental setup 2: Microtensile tests in osmotic liquid

This second setup has been developed to allow tensile tests in liquid medium with simultaneous strain measurements directly on the sample [3]. In principle strain measurements are not only possible in the longitudinal but also in the transverse direction.

During sample mounting the tester is in a horizontal position. The hypocotyls were mounted in between silicon half-pipes and smaller pipes fitting the groove of the larger half-pipe and fixed with a small amount of cyan-acrylate glue (Fig 1, vertical hypocotyl test). The pressure exerted by the metal plates of the tensile tester is distributed by the silicone on glue and hypocotyl, which allows instantaneous glue-hardening without damage of the hypocotyl. However, this clamping does not allow for any sample rotation (red arrow in Fig 1). To facilitate strain measurements on the sample, plants were carefully marked with a permanent marker in two positions spaced apart as far as possible. Afterwards the tensile tester was moved into a vertical position. A cuvette (optical glass) filled with an osmotic liquid (0.379 M Polyethylene glycol 200 solution) was imposed to prevent the hypocotyls from drying and to ensure stable optical conditions. Illumination both in transmission and reflection is a pre-condition for appropriate image quality. The hypocotyls were strained (approximately 3–5 min after harvesting) with a constant speed of 10 μm/s. Simultaneously, images of the sample were captured with a camera through a macroscope, and the force was recorded by a highly sensitive load cell with a maximum capacity of 500 mN (Honeywell Sensotec, Model 31E). Subsequent strain analysis relating time, force and displacement were performed in LabView.

### Calculation of mechanical parameters

The measured forces F during the mechanical experiments were converted into stresses σ with the following relationship σ = F/A, where A is the cross sectional area of the hypocotyl. The strain *ε* is defined as the change in length Δl divided by the initial length l_0_ (ε = Δl/l_0_). From the resulting stress-strain-diagrams (diagram in Fig 1) tensile stiffness and ultimate tensile stress σ_max_ (or fracture stress) were determined. The tensile stiffness E describes the resistance of a material or structure against elastic deformation and is defined as the quotient of tensile stress σ and strain *ε* in the linear elastic region of the stress-strain curve.

The reduction in hypocotyl diameter during tensing in the submerged setup was determined. For this, the diameters of the hypocotyls were measured on three positions of each hypocotyl before force was exerted and then again at the point of maximal force for hypocotyls of each tested age (n>30).

### Hypocotyl dry weight/wet volume

The cell wall proportion in a given cross section of the hypocotyl influences the mechanical properties. Hence a mean value of 3–5 hypocotyls for the dry cell wall mass per wet volume (a measure for “density”) was determined. Lengths of freshly harvested hypocotyls were measured by placing them under a macroscope equipped with a camera for capturing images. Afterwards cotyledons including the upper part of the stem and roots were excised and an image of the remaining cylinder was taken. Both hypocotyl length and volume assuming a cylindrical geometry were determined with ImageJ (Rasband, W.S., ImageJ, U. S. National Institutes of Health, Bethesda, Maryland, USA, http://imagej.nih.gov/ij/, 1997–2014). Afterwards 3–5 hypocotyls were packed in calibrated small and closable aluminium cups and dried for 24 h. After reaching a state of equilibrium (dry hypocotyl mass) the cups were weighed with an ultra-micro balance (Mettler Toledo, XP6U).

### Cellulose content

Fresh plant material was harvested in 2 mL Eppendorf reaction tubes. Seedlings stored in ethanol were transferred to a 2ml Eppendorf tube and air-dried overnight. Steel balls, 3 mm in size, were added and seedlings directly frozen in liquid nitrogen. The tissue was homogenized with a Retsch Mill for 2 min at 25–30 Hz. The powdered plant material was washed with 70% aqueous ethanol (v:v), steel balls were removed and samples spun down for 10 min at 14000 rpm in a centrifuge. After centrifugation the washing step with ethanol was repeated. The supernatant was discarded and after air drying the pellet, 1 mL of methanol: chloroform (1:1 [v/v]) was added and sonicated for 10 minutes. The samples were shaken manually and spun for 10 min at 14000 rpm again. The supernatant was discarded and the remaining pellet was washed twice with acetone and air dried for 1 hour to obtain cell wall material.

Dry cell wall material (0,5–1 mg) was transferred into a screw capped Eppendorf tube. Trifluoroacetic acid (TFA, 2 M, 250μl) was added and incubated for 90 minutes at 121°C in a heating block. After adding 300 μl isopropanol, the acidified TFA solution was evaporated under a stream of dried air. To help the elimination of the leaving acid by evaporation, repetitive addition of isopropanol was necessary. The hydrolysed material was suspended in water (200 μl) and centrifuged for 5 minutes at 13 000 rpm. The supernatant was removed and the insoluble pellet was considered for cellulose analysis. The cellulose content was determined using a modified protocol of [19]. The insoluble TFA-hydrolysed pellet was mixed with 175 μl of 72% sulphuric acid. The samples were shaken for 30 minutes at room temperature, sonicated for 15 minutes and shaken again for 15 minutes and mixed with 425 μl of water. After centrifugation, 200 μl of the samples were transferred to a new reaction tube and the glucose content of the solubilised material was determined using the anthron assay. 500 μl of anthrone reagent (0.2% [w/v] anthrone in concentrated H_2_SO_4_) was added. The mix was vortexed and boiled for 5 minutes. After cooling down, a portion of the sample (200 μL) was transferred to a 96-well microtiter plate and the absorbance was recorded at a wavelength of 640 nm using a Spectra Max Plus ELISA reader (MolecularDevices, Munich, Germany). A standard curve was prepared using glucose in a range of 0 μg to 6 μg.

## Results

### Hypocotyl geometry

**Fig 2:**
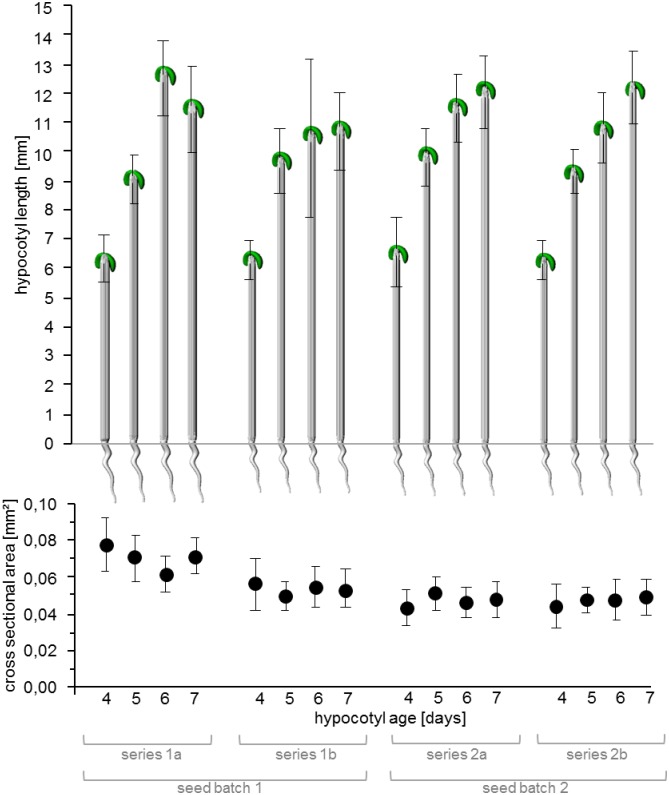
Hypocotyl lengths and cross sections of samples tested with experimental setup 1 (horizontal test, water vapour). For each series n > 18 replicas were analyzed. Error bars depict standard deviation.

Hypocotyl length increases with age, while the absolute increase per day is decreasing over time (Fig 2). The hypocotyl cross-sections remained almost constant over time within a particular test series.

**Fig 3:**
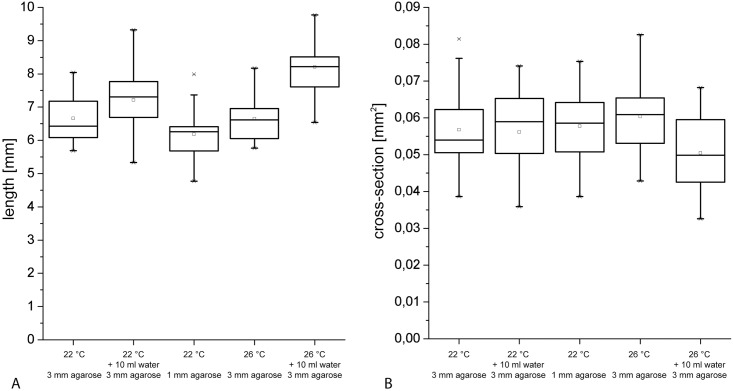
Influence of variations in growth conditions on the geometry of four day old dark grown hypocotyls. A) Length vs. growth conditions. Increased water content in the medium as well as a higher temperature during germination leads to an increase in length. A thinner layer of agarose appears to reduce the nutrient supply sufficiently to slightly decrease length growth. B) Cross-sections vs. growth conditions. Only the combination of an increased temperature during germination and higher water content in the medium induced a slight reduction in cross-section, while the individual alterations and a reduction in the amount of medium had no effect.

The cross sections of series 1a (Fig 2) were larger than all other tested hypocotyls. Hypocotyl cross section was tested against the seed series in a two-factor ANOVA including seed series and age as factors. The P-value for the factor seed series was highly significant (< 10^−16^) when series 1a was considered and substantially lower, when it was excluded from the test (6*10^−5^). The age tested over series 1b, 2a and 2b does not have a systematic effect (P = 0.41). However, this is not the case for series 1a where the age appears to have an influence on the hypocotyl cross-section (P = 0.002). To exclude the possibility that deviations in cross-section occurred due to variations in growth conditions, 4 day old plants were grown in the following alterations of the growth conditions compared to the standard conditions: a) less medium (only 1 mm of agarose on the plate compared to 3–5 mm of agarose in standard conditions), b) addition of 10 ml of water to the standard medium on each plate, c) an increase in growth temperature from 22°C to 26°C and d) a combination of an increase in temperature and additional water (Fig 3). Elevated temperatures (22°C to 26°C) and increased water-content in the medium were both favourable for elongation growth with cooperativity of the conditions. The hypocotyl cross section on the other hand was much more robust to alterations in growth conditions. Only the combination of an increased temperature and a higher water-content in the medium induced a slight decrease in cross-section.

### Density and cellulose content

**Fig 4:**
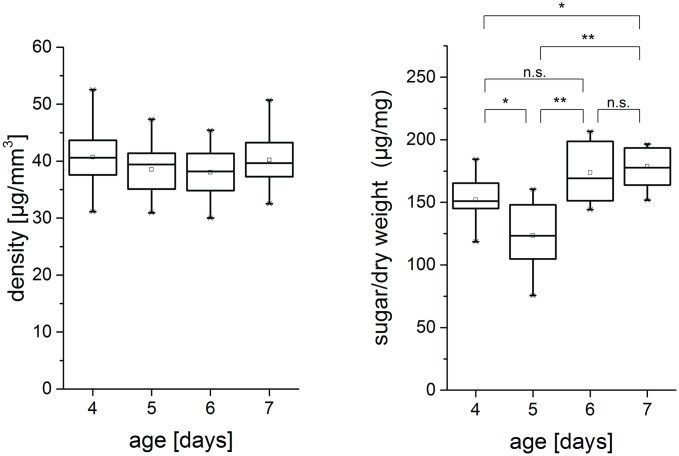
Density and cellulose content of dark grown hypocotyls. A) Density is analyzed as dry weight per wet volume (seed batch 1, n > 14). B) Cellulose content measured as μg hexoses per cell wall material (CWM). * P < 0.05, ** P < 0.01, n.s.: no significant difference.

The mechanical properties of hypocotyls depend not only on geometry but also on the density, measured as dry weight per wet volume. On average a density value of 39.69 ± 4.56 μg/mm^3^ was obtained for seed batch 1 which does not change significantly between four to seven day old plants (Fig 4A; ANOVA: P = 0.64). A non-parametric Mann-Whitney-Test showed that the cellulose content decreases significantly from four to five day old plants and then increases again with hypocotyl age. While the difference between five and six day old plants is significant, there is no significant difference between six and seven day old plants. All significances are measured at the 0.05 level.

### The mechanical parameters

**Fig 5:**
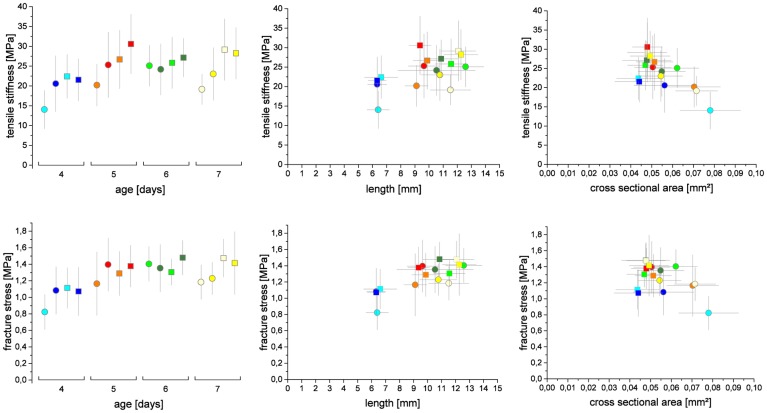
Tensile stiffness and fracture stress of series 1a (round, light symbols), 1b (round, dark symbols), 2a (square, light symbols, 2b (square, dark symbols). Plotted against age, lengths and cross sectional areas. For each set n>18 samples were analyzed respectively.

Mechanical properties were determined on the basis of hypocotyl cross-sections for two seed batches of Col-0 in the horizontal testing setup. For each seed batch 2 test series (a and b) were performed. Tensile stiffness ranges from 14.0 (+/- 4.8) to 25.3 (+/- 5.2) MPa and the fracture stress σ_max_ from 0.82 (+/-0.21) to 1.4 (+/- 0.21) MPa. Fig 5 shows means and standard deviations of all 4 test series of 4, 5, 6 and 7 day old hypocotyls. ANOVA reveals a highly significant difference (P = 10^−9^) when age is compared to tensile stiffness. However, when the four-day-old hypocotyls are excluded from the analysis, no significant difference (P = 0.7) can be found between the remaining time points. Likewise, the values for fracture stress are significantly different with regard to age (P = 10^−15^), yet no significant differences were found when only hypocotyls of 5-7-days of age were considered (P = 0.2).

### Evaluation of a tensile testing setup with submerged samples

**Fig 6:**
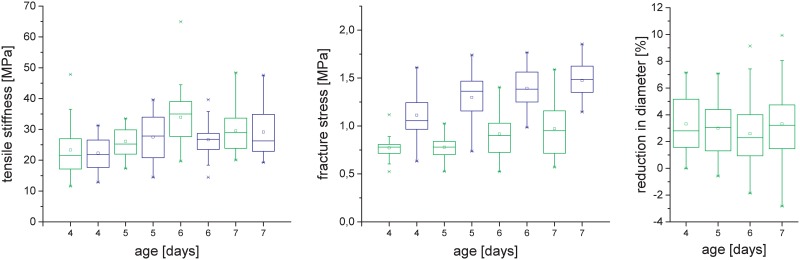
Comparison of the results of the micromechanical tests with samples tested submerged in an osmotic liquid (green, Setup 2) and in air, kept in the native state by a humidifier (blue, Setup 1) for tensile stiffness (A) and fracture stress (B). The change in diameter from the initial value before the tensing to the point of maximal stress in the submerged setup (C).

In addition to the first setup in which samples were tested in humidified air, a second setup with more advanced options of strain measurements and of tuning the testing conditions by variation of the liquid medium in which the samples are submerged was applied. We evaluated whether the obtained mechanical parameters are comparable to the standard setup 1. For this, the tensile tests of the second seed batch were also performed in liquid (Setup 2). The mechanical parameters obtained by the two micromechanical tensile testers show very similar values for tensile stiffness (Fig 6A). This was tested in an ANOVA where a P-value of P = 0.04 was found. The fracture stress was significantly lower (P = 10^−16^) for all samples tested in the second setup in liquid medium than for those tested in humidified air (Fig 6B). For all mechanical tests tensile stiffness was calculated based on hypocotyl cross sectional area before the test (the so-called engineering stress). Since many materials reduce their cross sectional area during stretching we analyzed how diameters of hypocotyls changed during stretching. Hypocotyl diameter at maximum stress was reduced by 2–3% with no statistically significant difference related to hypocotyl age (Fig 6C).

## Discussion

In this work the mechanical properties—tensile stiffness and fracture stress—of the fully elongated zone of dark grown, living *Arabidopsis* hypocotyls were studied. The aim was to describe their changes with time and how these changes relate to hypocotyl geometry, density and cellulose content. Besides getting answers to the question of how the mechanical properties of *Arabidopsis* hypocotyls change with time, it was also important for us to establish reliable test conditions for future experiments on modified *Arabidopsis* hypocotyls.

Length of the hypocotyls increased with age, however growth slowed down within the analyzed time span. This is consistent with results from [5]. A slight variation of hypocotyl diameter was found between series 1b, 2a and 2b while the hypocotyl diameters of series 1a were considerably larger, underlining the importance of recording geometrical parameters for each individual test series. The hypocotyl diameter within test series 1b, 2a and 2b remained almost constant, which is also in agreement with literature values [15]. Surprisingly, we found a high variation in the cross sectional area between the two series out of seed batch 1, while the hypocotyl lengths were similar. Since the reason for this was unclear, a follow-up experiment testing the effect of varied growth conditions on cross-section and length was performed. It could be excluded that environmental variation caused the observed effect. While the length could be influenced by alterations in growth conditions, the cross section appeared more robust. Thus, altered conditions during sowing as well as an altered procedure of the experimenter are unlikely to account for the variations observed but cannot be completely excluded. The experimenters were already experienced with the setup when the tests were performed so that we are confident that it was not a variation in handling that caused the effect. When using Col-0 as a reference system to study the effect of a treatment or mutation on hypocotyl mechanics, the unexpected variation in hypocotyl diameter stress the importance of at least two replica measurements even under controlled growth and test conditions.

Density is a parameter closely related to the amount of cell wall in a given hypocotyl volume. No significant changes could be observed from 4–7 day old plants for seed batch 1. This implies that the cell wall proportion, in this case, is not a significant factor that influences the mechanical parameters. Furthermore, density could not be determined on individual hypocotyls but on batches of 3–5 hypocotyls so that only average values were obtained. For these reasons, it was decided to calculate stresses by the division of the force through hypocotyl cross-section rather than by division through the cell wall proportion. This has the advantage that strain determination is based on the measurement of the cross-section of the very same sample that is tested in tension rather than on averaged density values. For the given reasons, the parameter was not considered in seed batch 2. Although density remains constant, the cellulose content drops from the fourth to the fifth day of growth and then increases again. This decrease in cellulose content coincides with the time-point of the largest length increase, which indicates reorganization of the cell wall [15].

The experimental data of all four test series show that tensile stiffness and fracture stress of four day old hypocotyls is always lower compared to five to seven day old hypocotyls. This finding indicates that elongation processes are not fully completed in the analyzed basal region four days after sowing. Relaxation processes in the cell wall or between cells or incorporation of matrix substances [20] could alter cell wall mechanics just like changes in microfibril arrangement [21, 22] and cellulose synthesis. The cellulose content decreases during the phase of large elongation from day four to day five (Fig 4), while the mechanical properties reach a level similar to that of six and seven day old plants. This could indicate that the amount of material plays a minor role for the mechanical properties at this growth stage while other factors like the structural organization of the microfibrils might be of higher relevance.

Hypocotyl age, defined by the time after sowing, includes uncertainties concerning germination time, therefore uncertainties concerning the developmental stage, hypocotyl length and cross section have been determined additionally prior to each tensile test. The detected good correlation between hypocotyl length and age (Fig 2) simplifies sampling procedures: it allows the experimenter to choose if age and/or hypocotyl length is the main criterion for sampling. However, one explanation for the outliers in the data such as the data point of series 1a for 6 day old plants, could be a bias of the experimenter during sampling just like samples growing differently on a particular plate. In terms of dark-grown hypocotyls time after sowing might be the more practical parameter compared to hypocotyl length, which can be difficult to monitor while ensuring complete darkness during growth. However, additional knowledge about hypocotyl length might always be valuable to detect differences in germination time even though the variation in length between hypocotyls is high. Furthermore, datasets from experiments where only one of the two parameters was measured can be compared.

In the course of the presented work, a second setup was developed allowing measurements in liquid, which ensures stable optical conditions enabling strain-detection directly on the sample. This approach has the advantage to exclude any errors coming from the glue spots of the other tensile tester. Furthermore, it is possible to measure strains in the transverse direction as well as to test the influence of variations in the osmotic and enzymatic environment of the sample. For this paper, we tested how comparable the mechanical parameters tensile stiffness and stress at failure are between this setup with submerged samples and the setup in which hypocotyls are tested in humidified air. Our results showed that tensile stiffness is comparable between the two setups with very slight differences that can probably be accounted to an outlying measurement of the six day old hypocotyls highlighting the importance of replicas. Furthermore the results indicate that the longer sample preparation times of setup 1 do not (measurably) effect tensile stiffness of the hypocotyls. In contrast to tensile stiffness, stress at failure was considerably lower for the device with submerged samples. The reason for this is that in a tensile test a material tends to break at its weakest point, which can be either an imperfection or a region which experiences higher stresses than others. Non-tapered samples like the hypocotyl tend to break in regions with additional stresses besides pure tension, e.g. clamping sites. Whereas the foliar frames used in experimental setup 1 (Fig 1, horizontal test) allow for a certain sample rotation/alignment of the hypocotyls during testing, the clamping by the infusion pipes does not. This means that the stresses at the clamping sites in the experimental setup 2 (Fig 1, vertical test) are not only stresses in tension but superimposed by other stresses, e.g. bending moments which finally lead to fracture at lower force levels. However, this drawback has to be put in relation with the particular research question: The second setup has a high potential when strain behaviour in transverse and longitudinal direction are to be determined as well as when the control of the osmotic environment for the sample by different liquids or the influence of different enzymatic treatments is in the focus of the analysis. However, when stress at failure is the parameter of interest, the first setup is to be preferred. In principle our first setup is comparable to tensile testing setups being used in the past by other groups, e.g. [2, 9, 23]. Many of these experiments were performed on pre-treated plant systems to exclude effects coming from growth related processes such as changes in turgor pressure. This topic has been amply discussed in a review by Cosgrove [8]. Pre-treatments are in principle also possible for our systems. However, the experiments on hypocotyls with the second setup are very different to previously described tensile tests. Deformations and fracture processes in more than one dimension can be studied.

Growth of the plant cell wall has been described as a multi-step process in which cellulose synthesis and polymer integration is followed by a selective wall loosening and subsequent yielding of the cell wall. This stress relaxation then leads to a drop in turgor pressure which plants compensate for by water uptake, thereby restoring turgor pressure. Cessation of wall expansion is marked by an altered cell wall composition with an increased number of cross-links [24]. Within the measurable range, no significant changes in turgor pressure were observed [7], indicating that the amplitude of changes in turgor pressure during the described growth process are small or that averaging over the long measuring time spans necessary for the experiment prevented the detection of any differences. For this reason, turgor pressure analysis was not considered for this study.

Changes in the measured parameters in the course of the hypocotyl development from four to seven days after sowing were largest from day four to day five, indicating that a transition from one growth stage to the next takes place. On the one hand this has to be taken into account when analysing and comparing datasets of different origin, on the other hand there are questions arising as to the exact processes taking place during this time and how they are related to external conditions. The high variation found in the time series to determine the mechanical properties as well as morphological and physiological traits underlines the importance of a sufficiently large number of replicas of a sample grown under carefully chosen and controlled conditions. During the analysis of cell wall mutants this time point of large changes in hypocotyl development might be altered and is an important parameter when comparing data from mutants to those of wild-type plants. By comparing time courses of cell wall mutants to those of wild type plants, we might be able to distinguish between effects caused by a slower development and thus smaller seedling size and an altered mechanical response due to alterations in the cell wall ultrastructure.

The methods described enable us to follow the development of the hypocotyls on a morphological level by analysing alterations in cell wall mechanics following growth. Hence, we can draw conclusions on the consequences of different growth parameters on hypocotyl mechanics. The material characterization achieved in this way can complement the knowledge on biochemistry and genetics related to primary plant cell wall growth. The values for tensile stiffness, ultimate tensile stress, density and cellulose content can add to the understanding of cell wall growth processes as they can be the basis for modelling approaches describing and comparing different cell growth scenarios.

## Conclusion

Two complementary protocols for the determination of the mechanical properties of hypocotyls have been developed. The proposed tools and the obtained data may serve as a reference and add to the understanding of hypocotyl growth and primary cell wall mechanics. Furthermore, they allow an estimation of inherent (biological) variability and the applicability of this approach. We assessed the influence of physiological and environmental parameters on hypocotyl growth and mechanics and determined the hypocotyl age that is most suitable for analysis. 5–7 days after sowing, the hypocotyls showed rather stable properties concerning hypocotyl diameter, density and tissue mechanics. Our data show that it is possible to use hypocotyl length as well as age as a parameter for the sampling process.

The presented approach has the potential to be highly useful for any group being interested in the characterization of *Arabidopsis* phenotypes regarding the impact of modifications and mutations on hypocotyl structure and mechanical properties.
